# Correction: Khort et al. Automated Mobile Hot Mist Generator: A Quest for Effectiveness in Fruit Horticulture. *Sensors* 2022, *22*, 3164

**DOI:** 10.3390/s25092643

**Published:** 2025-04-22

**Authors:** Dmitriy Khort, Alexey Kutyrev, Nikolay Kiktev, Taras Hutsol, Szymon Glowacki, Maciej Kuboń, Tomasz Nurek, Anatolii Rud, Zofia Gródek-Szostak

**Affiliations:** 1 Department of Technologies and Machines for Horticulture, Viticulture and Nursery, Federal Scientific Agroengineering Center VIM, 1-st Institutsky Proezd, 5, 109428 Moscow, Russia; dmitriyhort@mail.ru (D.K.); alexeykutyrev@gmail.com (A.K.); 2 Department of Intelligent Technologies, Taras Shevchenko National University of Kyiv, Volodymyrs’ka Str., 64/13, 01601 Kyiv, Ukraine; 3 Department of Automation and Robotic Systems, National University of Life and Environmental Sciences of Ukraine, Heroiv Oborony Str., 03041 Kyiv, Ukraine; 4 Department of Mechanics and Agroecosystems Engineering, Polissia National University, Stary Boulevard, 7, 10008 Zhytomyr, Ukraine; 5 Department of Machine Use in Agriculture, Dmytro Motornyi Tavria State Agrotechnological University, B. Khmelnytsky Ave., 18, 72312 Melitopol, Ukraine; 6 Department of Fundamentals of Engineering and Power Engineering, Institute of Mechanical Engineering, Warsaw University of Life Sciences (SGGW), 02-787 Warsaw, Poland; 7 Department of Production Engineering, Logistics and Applied Computer Science, Faculty of Production and Power Engineering, University of Agriculture in Krakow, Balicka 116B, 30-149 Krakow, Poland; maciej.kubon@urk.edu.pl; 8 Eastern European State College of Higher Education in Przemysl, Ksiazat Lubomirskich 6, 37-700 Przemysl, Poland; 9 Department of Biosystem Engineering, Institute of Mechanical Engineering, Warsaw University of Life Sciences (SGGW), 02-787 Warsaw, Poland; tomasz_nurek@sggw.pl; 10 Faculty of Engineering and Technology, Higher Educational Institution “Podillia State University”, 32316 Kamianets-Podilskyi, Ukraine; anatoliyrudj@gmail.com; 11 Department of Economics and Enterprise Organization, Cracow University of Economics, 31-510 Krakow, Poland; grodekz@uek.krakow.pl

In the original publication [1], there was a mistake in Figure 5. The subfigure “Account card 22” was wrongly printed. The correct Figure 5 appears below.

The authors state that the scientific conclusions are unaffected. This correction was approved by the Academic Editor. The original publication has also been updated.

## Figures and Tables

**Figure 5:**
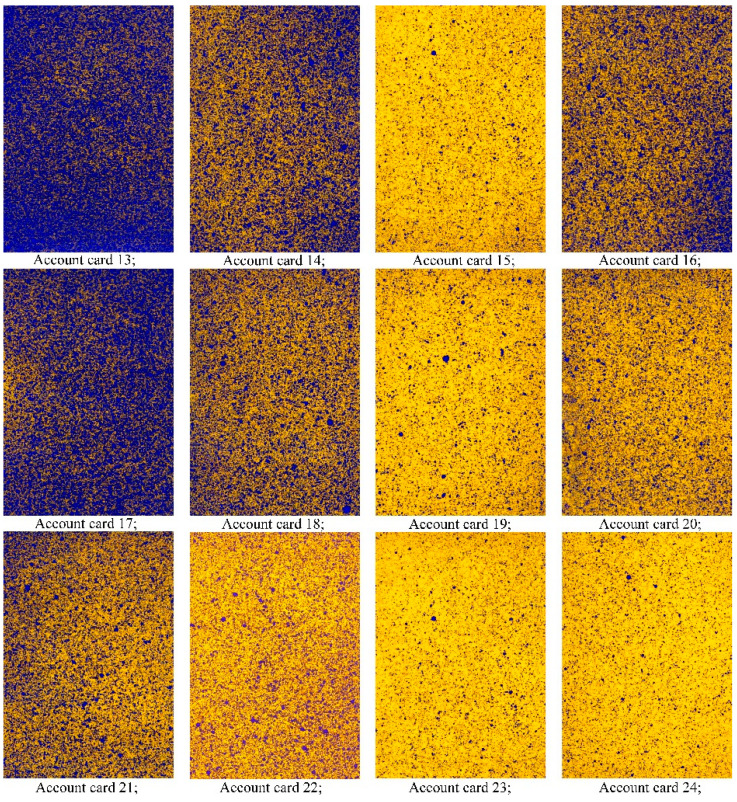
Account registration cards of the middle tier at a platform speed of 1.5 km/h.
